# Molecular Dynamics Simulation of Ti Metal Cutting Using a TiN:Ag Self-Lubricating Coated Tool

**DOI:** 10.3390/ma16041344

**Published:** 2023-02-05

**Authors:** Veniero Lenzi, Luís Marques

**Affiliations:** 1 Center of Physics of Minho and Porto Universities, University of Minho, Campus de Gualtar, 4710-057 Braga, Portugal; 2 Laboratory of Physics for Materials and Emergent Technologies, LapMET, University of Minho, 4710-057 Braga, Portugal

**Keywords:** molecular dynamics, nanocomposite coatings, self-lubricating coatings, cutting, wear

## Abstract

Silver-ceramic nanocomposite coatings, such as TiN:Ag, are among the most interesting solutions to improve the machining and cutting process of hard-to-cut Ti alloys, since they combine the TiN matrix hardness with the lubricating and protective action of Ag nanoparticles. Therefore, it is important to understand how, when present, Ag distributes at the tool-workpiece interface and how it affects the tribolayer formation and the tool wear. Molecular dynamics simulation results, obtained using a MEAM-based force field, are presented here for the cutting process of a Ti workpiece with a TiN tool, with and without the presence of Ag at the interface, for different cutting speeds. Ag is shown to form a thin protective layer at the workpiece-tool interface that prevents a direct contact between the parts and greatly reduces the tool degradation. Our simulations confirm the importance of Ag in self-lubricating nanocomposite coatings to realize the machining of otherwise hard-to-cut materials.

## 1. Introduction

Self-lubricating nanocomposite coatings with a solid lubricating agent are emerging as a promising technology to improve the machining and cutting of hard-to-cut materials, such as titanium-based alloys employed in aerospace industry [1]. Compared to traditional solutions, self-lubricating nanocomposite coating can lead to the reduction in lubricant usage and extension of the lifetime of cutting tools, thus leading to an increased environmental and economic sustainability [2]. Silver, in particular, is one of the most popular choices as lubricating agent [3,4,5,6,7,8], due to its softness, low melting temperature, and low affinity with transition metal carbides and nitrides [9,10].

In particular, ceramic-Ag nanocomposite coatings [11] have been proposed as an effective lubricating action while retaining a high degree of hardness. This is especially true with titanium nitride-based superhard coatings [12], in which silver is present in the polycrystalline TiN matrix as well-separated Ag nanoparticles [13], and a control of its release is crucial to obtain a constant and long-lasting lubrication. TiN:Ag coatings, with the inclusion of SiN in the matrix [14] and in multilayered architectures, have shown very good performance in terms of lubrication and wear protection [15,16]. To improve the performance of these coatings, which strongly depends upon their structure, and thus, on the Ag release rate, a deeper understanding of the Ag lubricating and protective action during the cutting/machining process is required.

Self-lubricating coatings could be effectively employed in micro- and nanomachining processes [17], whose importance is continuously growing due to the demand of miniaturization in industry [18]. Compared to other technologies, micro- and nanomachining is more versatile and material-efficient [19], and ceramic coatings are known to improve wear resistance and cutting performance of tools [20]. The small length and time scales of micro- and nanocutting processes when compared to their macroscale counterparts makes them suitable systems for molecular dynamic (MD) simulations, allowing for the study of the physical and chemical mechanisms to occur at the tool/workpiece interface at the atomistic level. While other simulation approaches, such as finite element simulations [21], allow for a large-scale simulation of the cutting process, MD allows for the tracking of forces, stresses, and temperature at the atomistic level, as well as the microscopic deformations of the tool/workpiece pair and, if a suitable forcefield is used, the tribochemical reactions at the interface. Therefore, MD simulations are now regarded as one of the key methods in understanding micro-and nanomachining, as testified by various comprehensive accounts [22,23,24].

Clearly, time and length scale limitations inherent to MD simulation techniques imply that only nanometric cutting [25,26] experiments can be directly compared and combined with MD simulations, as recently carried out by Liu et al. [27]. Nevertheless, simulation results are accurate in describing the local processes, and general conclusions can still be drawn. MD simulations have been extensively used to study the machining of silicon [28], revealing that its brittleness is related to the high confinement of plastic deformations [29] and specific conditions are required to effectively machine it [30]. Various other materials have also been studied including, among others, silicon carbide [31], copper [32,33], and ferrite [34]. Computational studies investigated the influence of cutting parameters on the process, such as the rake angle [35], cut depth and tool edge radius, [36], and temperature distribution as a function of cutting speed [37]. Further studies focused on the material removal mechanism, revealing that in nanocutting there are two possibilities; namely, a shear mode [38] that is comparable with what happens in macro-scale cutting [39], and an extrusion mode [40], in which the large stresses exerted by the tool on the workpiece induce a local amorphous phase transition and the formation of an amorphous chip. Simulations have also been used to investigate how the subsurface deformations of the workpiece are influenced by the cutting parameters [41,42]. Concerning tool wear, MD simulations demonstrated that, in case of diamond tools, the degradation is caused by the heat generated during the cutting [43], and other mechanisms are also relevant, such as diffusion wear [44] and graphitization [45]. Therefore, this brief overview demonstrates the relevance and validity of MD simulations in studying micro- and nanomachining processes.

To deal with different materials, the tool-workpiece interaction is typically modeled using generic pair potentials, such as Morse or Lennard-Jones. However, this approach hinders the investigation of tool wear since it greatly approximates the tribochemical processes at the interface, such as species interdiffusion and adhesion. As recently demonstrated in a MD study of the cutting of Ti by a diamond tip, the use of reactive force field is fundamental to correctly take into account these phenomena [46]. When a lubricating agent is present, such as Ag, a consistent modeling of interactions between the different species is fundamental in order to capture its effect in the cutting process.

The aim of this work is to understand the lubricating and protective role of Ag at the tool/workpiece interface in the nanomachining of a pure Ti workpiece with a TiN tool. To achieve this, we performed MD simulations of the cutting process using a MEAM force-field specifically developed for TiN:Ag systems [47], which embodies the tool-workpiece interaction and thus allows for an effective simulation of the wear phenomena, including interdiffusion and tool-workpiece-lubricant reactions. The cutting process will be studied at different cutting speeds, in order to characterize the different regimes. This paper assesses for the first time the effects of Ag solid lubricant in the nanomachining of a Ti surface using a TiN-coated tool using MD simulations.

## 2. Materials and Methods

All molecular dynamic simulations have been performed using LAMMPS, 29 September 2021 version [48]. Interatomic forces have been modeled with the hybrid MEAM-Mie force field for TiN:Ag systems. In particular, we combined the existing MEAM forcefields for Ti-Ag [49] and TiN [50] systems and included a purely repulsive Mie pair potential [51] to consider Ag-N interactions, since no stable Ag-N compounds exist to allow for the MEAM potential parametrization for this pair. Further details on the forcefield development and validation can be found in Ref. [47].

**Figure 1:**
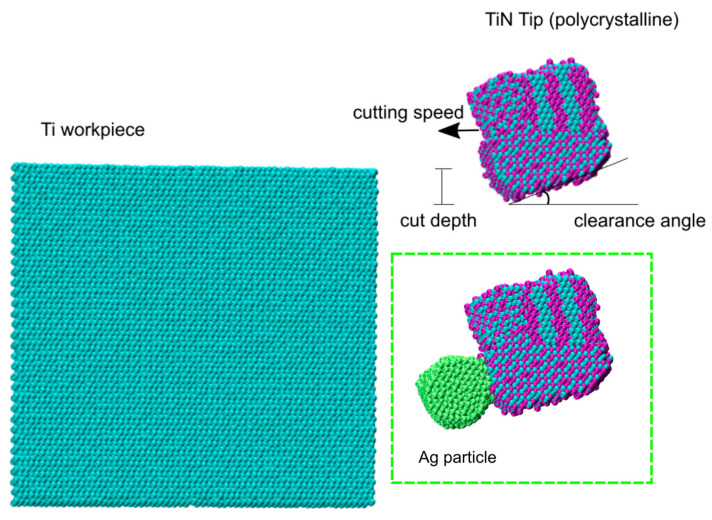
Initial configuration for the cutting simulation. Inset shows the TiN tip with the Ag particle added. In this picture and in the following, Ti atoms are cyan, N atoms are purple, and Ag atoms are green.

In all simulations, a timestep of 1 fs was always used. The cutting tool was modeled as a polycrystalline 6 × 5 × 5 nm^3^ stoichiometric TiN block, and the workpiece was represented by 6 × 17 × 14 nm^3^ Ti monolithic block. Both the tool and the workpiece have been subject to an energy minimization step to remove unwanted overlap and then annealed under NPT conditions for 500 ps at 900 K and 0 atm, followed by a 500 ps equilibration step at 300 K and 0 atm. The NPT conditions were enforced by a Nosé-Hoover thermo- and barostat [52,53], with damping constants of 0.1 and 1 ps, respectively. After this step, the tool and workpiece were assembled in a simulation box measuring 6 × 30 × 30 nm^3^, as shown in Figure 1.

For the cutting process, a clearance angle of 10 degrees was considered, and the cut depth has been selected as 1.8 nm. To keep the workpiece in place during the cutting process, a narrow strip of workpiece atoms at the simulation box boundary was kept frozen, and another neighboring strip of atoms was thermostated at 300 K to enable excess heat dissipation [28]. The same has been carried out with the cutting tool, i.e., freezing and thermostating the tool’s outer atomic layers on the sides opposite to the contact area. The presence of silver has been simulated by generating a nanoparticle of about 2000 Ag atoms in correspondence of the tool surface and allowing it to equilibrate with the tool. In this way, the effect of presence of Ag at the tribolayer can be readily investigated.

All the other atoms were simulated under NVE conditions. To realize the cutting process, a cutting speed V was imposed on the frozen atoms of the tool and remained constant throughout the simulation. We considered three cutting speeds of 1, 10, and 100 m/s. While the first two are compatible with speeds used experimentally for high-speed cutting of Ti alloys, the latter speed allows for the investigation of the condition in which the cutting timescale is larger than the temperature and stress relaxations times in the workpiece The simulation has been performed until the cutting tool reached the simulation box wall. During the simulation, snapshots were saved at regular intervals, in order to be post-processed for further analysis. In particular, Von Mises stress and temperature, the latter being estimated from the atomistic kinetic energy, were calculated for every atom of the system by an average over a time window of 0.1 ps. The cutting force *F_c_* was obtained by summing the atomistic forces of the atoms constituting the tool, and averaging the results over a time window of 1 ps. To compare the simulations at different speeds, results are presented as a function of the (time dependent) cut distance, defined as the distance traveled by the cutting tool in a specific instant, measured from the workpiece rightmost edge.

The polycrystalline structure of the tool was created using atomsk, version b0.11.2 [54]. All graphical representations were obtained using VMD, version 1.9.3 [55].

## 3. Results

### 3.1. Unlubricated Cutting

**Figure 2:**
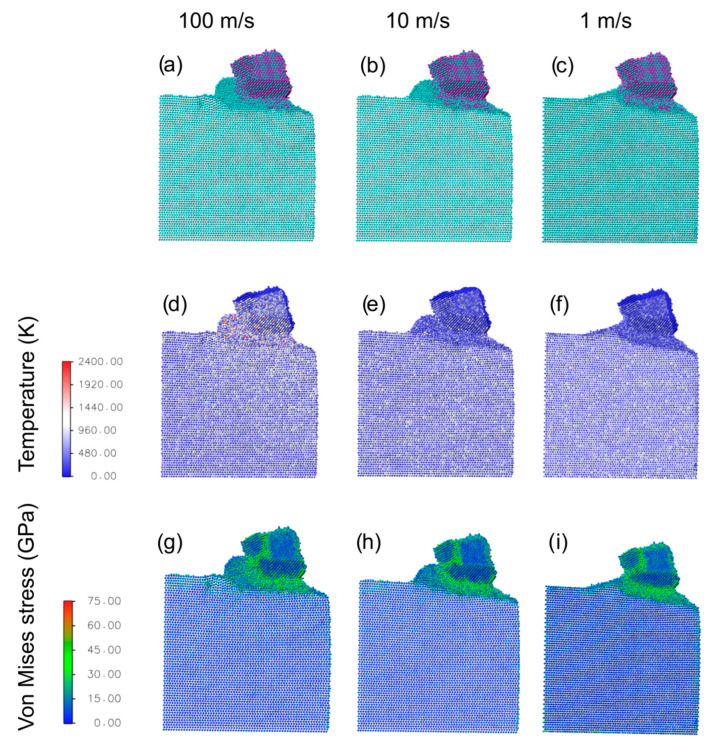
MD results for the cutting process, taken at a cut distance of 7 nm in the unlubricated case, as a function of velocity (each column refers to the value reported on top). Panels (**a**–**c**) report simulation snapshots, panels (**d**–**f**) temperature maps, and panels (**g**–**i**) Von Mises stress maps.

Due to the high affinity between Ti and TiN, we expect that the cutting of a Ti workpiece with a TiN-coated tool is an extremely hard process at any cutting speed. To confirm this, we conducted cutting simulation with the bare (i.e., without Ag lubricant) TiN tip at three different speeds of 100, 10, and 1 m/s. Snapshots taken at the same cut distance of 7 nm at the 3 speeds considered are reported in Figure 2a–c, along with their respective temperature (Figure 2d–f) and Von Mises stress (Figure 2g–k) atomistic maps.

The cutting speeds affect the dynamics of the cutting process. At the highest cutting speed of 100 m/s, the heat and stress generated by the tool do not have sufficient time to relax. Therefore, the contact region below and in front of the tool are amorphous, indicating a local melting. At the lower cutting speed of 10 m/s, the size of the amorphous contact region is considerably reduced. This is reflected by the shape of the chip, which shows a smoother contact with the uncut region compared to the fast cutting case. At the slowest cutting speed of 1 m/s, owing to the fact that stresses and temperatures have sufficient time to relax, the cutting process is completely different: The chip undergoes a full plastic reconstruction, with the formation of coherent crystal planes in the workpiece, specifically in front of the cutting tool, while the amorphous region is limited to a few atom-thick layers at the workpiece-tool interface. We can conclude that at the highest cutting speed the material removal occurs through an extrusion process, while at lower speeds it occurs through shear [38,40]. Regarding the tool wear, at all speeds, a significant tool degradation can be seen when compared to the initial state depicted in Figure 1, regardless of the cutting speed. The tool region corresponding to the cut depth is rendered amorphous during the process, which results in a lower effective cut depth. Moreover, a trail of leftover material is clearly visible, which appears to merge with the workpiece surface. Specifically, there is a significant incorporation of N atoms on the workpiece surface after the passage of the tool.

Regarding temperatures (Figure 2d–f), we can see that the generated heat during the process is localized in the tool and in the chip. The highest cutting speed results in larger temperature when compared to the other cases. Finally, The Von Mises stress distribution (Figure 2g–i) shows that the mostly stressed regions are at the forefront of the tip and under it, i.e., in correspondence of the amorphous TiN layer formed during the cutting process. Within the tip, the grain boundaries are subject to large stresses. No appreciable differences can be found between different cutting speeds.

### 3.2. Ag-Lubricated Cutting

**Figure 3:**
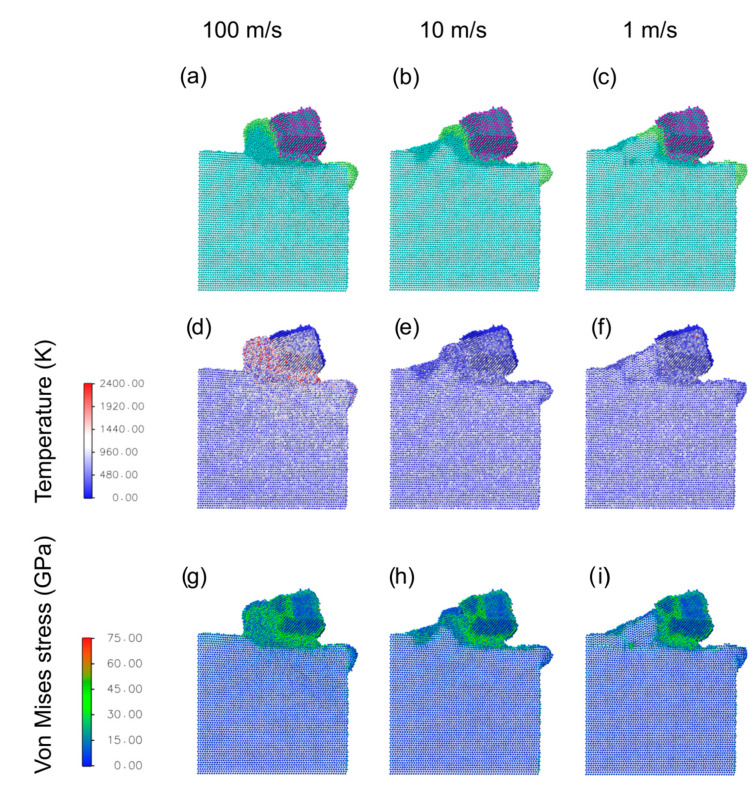
MD results for the cutting process, taken at a cut distance of 7 nm, with the presence of silver at the interface, as a function of velocity (each column refers to the value reported on top). Panels (**a**–**c**) report simulation snapshots, panels (**d**–**f**) temperature maps, and panels (**g**–**i**) Von Mises stress maps.

Figure 3 reports a snapshot of the cutting process when a Ag nanoparticle is present at the tool/workpiece interface, along with temperature and Von Mises stress maps, as a function of cutting speed. It is immediately evident that Ag provides a significant protective action from wear, by forming a thin layer at the tool/workpiece contact region. This prevents their direct contact and greatly reduces the tribochemical reactions. Compared to the unlubricated cut case (Figure 2), the lower region of the tool in contact with the workpiece is significantly more preserved at every speed (Figure 3a–c), and a clean cut is obtained. The previously noted incorporation of N atoms within the workpiece is now almost absent. Considering that the cut depth is the same for the unlubricated and ag-lubricated simulations, the lower tool degradation in the latter case indicates that a larger effective cut depth is reached in this case, resulting in larger chips. Regarding the chips’ morphology and material removal mechanism, we observe similar trends with respect to the cutting speed as the unlubricated case, with an amorphous liquid-like chip and an extrusion process observed at 100 m/s. At 10 m/s the chip amorphous region is greatly reduced, while reconstruction can be seen at the forefront of the cutting tool. At the slowest cutting speed of 1 m/s, the Ti workpiece reacts to the cut by accumulating material in front of the cutting tool and undergoing an evident restructuring, with no amorphous chip formation. The formation of grains with different orientations, as a reaction to accommodate the stress induced by the tool, can be observed. Notably, in all cases, Ag creates an interfacial layer between the chip and the tool, which is expected to greatly help the tool detachment from the workpiece, due to the low affinity between Ag and TiN.

By analyzing the temperature maps, we can observe a significant heating only at the highest speed (Figure 3d), in correspondence of the contact region and the chip, in agreement with previous works [37], while no large change in temperature can be observed at lower speeds (Figure 3e,f). The heating of Ag at the highest cutting speed suggests that it could play a role in transporting away the heat from the cutting region. Due to its low melting temperature, especially for small nanoparticles [56], Ag is expected to be in a liquid state, thus with very high mobility. This suggests that Ag-lubricated coatings could be combined with other lubrication techniques, such as the use of cryogenic and/or high-pressure coolants [57,58,59], provided that the coolant jet does not completely remove all the Ag at the interface. At the slowest cut speed, a coherent interface at the top of the chip between Ti and Ag can be seen to form.

From the Von Mises stress analysis (Figure 3g–i), the same conclusions as from the unlubricated case can be drawn. At the slowest cutting speed (Figure 3i), the cut-induced grain-boundaries in the Ti workpiece are highlighted by larger values of Von Mises stress.

### 3.3. Cutting Forces

**Figure 4:**
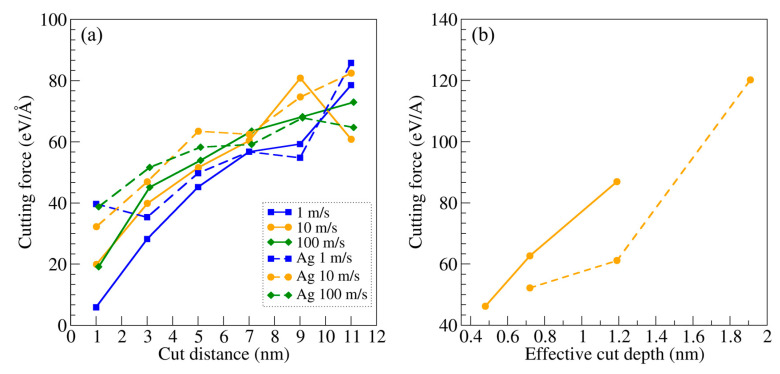
(**a**) Calculated cutting forces, for different cutting speeds, as a function of cut distance for the unlubricated (full lines) and lubricated (dashed lines) cutting simulations. (**b**) Calculated cutting force as a function of the effective cut depth. Legend is the same as in panel (**a**).

The cutting force *F_c_* evaluation is important to assess the lubricating effect of Ag. In Figure 4a, we report on the calculations for lubricated and unlubricated cuts and for all the cutting speeds considered to date. There are no significant differences between the curves, with a steady increasing trend as the cutting proceeds. At the highest cutting speed, the curve is smoother when compared with the others. This erratic behavior in cutting force at lower speeds might be due to the fact that plastic rearrangements and material accumulation occur in front of the cutting tool, locally altering the mechanical properties of the workpiece. Furthermore, the tool itself undergoes significant deformations and shows a large degree of adhesion with the workpiece, implying that a stationarity of the cutting force is unlikely to be reached, unlike as expected for different simulation setups [60]. This effect is particularly evident for the slowest cutting speed of 1 m/s, for which we observe a large accumulation of Ti in front of the tip and larger tool wear. As it can be appreciated in Figure 3, the effective cut depth is significantly larger in the case of Ag lubrication. This indicates that, even if *F_c_* is the same for lubricated and unlubricated cutting, the lubricated cutting is significantly more effective, thereby confirming the lubricating capability of Ag. To further validate this observation, we performed lubricated and unlubricated cutting simulations at the cutting speed of 10 m/s for different initial cut depths, i.e., 1.1 and 2.5 nm, and calculated *F_c_* at a cut distance of 7 nm as a function of the effective cut depth, which was considered as the average distance between the already cut and initial Ti surface. Results are reported in Figure 4b. In both lubricated and unlubricated processes, *F_c_* increases with the effective cut depth, as expected [36,60]. Regarding Ag, it is confirmed to improve the cutting process by enabling a larger cut depth.

## 4. Conclusions

We simulated the cutting process of a Ti workpiece with a TiN tool, for different simulated cutting speeds and, for the first time, evaluated the effects of the presence of Ag at the interface. Without any lubrication the cutting is basically unfeasible, as expected by considering the high affinity of Ti with TiN. The tool rapidly degrades and loses its cutting capability, regardless of the cutting speed considered. Conversely, when Ag is present, it forms a protective layer at the workpiece-tool interface that dramatically reduces the tool wear, basically rendering the cutting process possible. Moreover, Ag accumulates at the chip-workpiece interface, possibly suggesting a significantly easier tool detachment when compared to the unlubricated case. Furthermore, the lubricating capability was confirmed by the *F_c_* analysis.

The simulations presented here clearly represent a simplification of the actual cutting process and neglect many factors, such as the presence of oxygen and/or water, as well as more complex formulations for both the tool and the workpiece. Nevertheless, the cutting process, tool wear, and Ag effects were simulated in a fully consistent way. The use of a force field that allows for the treatment of the tribochemical phenomena at the interface allowed us to achieve a deeper understanding of the physical phenomena that occur between the workpiece and tool tip.

## Data Availability

Data supporting the results presented in this paper will be provided by the corresponding author, upon reasonable request.
